# A novel nomogram to predict the overall survival of early-stage hepatocellular carcinoma patients following ablation therapy

**DOI:** 10.3389/fonc.2024.1340286

**Published:** 2024-02-07

**Authors:** Honghai Zhang, Shugui Sheng, Wenying Qiao, Ming Han, Ronghua Jin

**Affiliations:** ^1^ Interventional Therapy Center for Oncology, Beijing You’an Hospital, Capital Medical University, Beijing, China; ^2^ Beijing Key Laboratory of Emerging Infectious Diseases, Institute of Infectious Diseases, Beijing Ditan Hospital, Capital Medical University, Beijing, China; ^3^ Beijing Institute of Infectious Diseases, Beijing, China; ^4^ National Center for Infectious Diseases, Beijing Ditan Hospital, Capital Medical University, Beijing, China; ^5^ Changping Laboratory, Beijing, China

**Keywords:** hepatocellular carcinoma, ablation, Lasso regression, multivariate Cox regression, overall survival, nomogram

## Abstract

**Introduction:**

This study aimed to assess factors affecting the prognosis of early-stage hepatocellular carcinoma (HCC) patients undergoing ablation therapy and create a nomogram for predicting their 3-, 5-, and 8-year overall survival (OS).

**Methods:**

The research included 881 early-stage HCC patients treated at Beijing You’an Hospital, affiliated with Capital Medical University, from 2014 to 2022. A nomogram was developed using independent prognostic factors identified by Lasso and multivariate Cox regression analyses. Its predictive performance was evaluated with concordance index (C-index), receiver operating characteristic curve (ROC), calibration curve, and decision curve analysis (DCA).

**Results:**

The study identified age, tumor number, tumor size, gamma-glutamyl transpeptidase (GGT), international normalized ratio (INR), and prealbumin (Palb) as independent prognostic risk factors. The nomogram achieved C-indices of 0.683 (primary cohort) and 0.652 (validation cohort), with Area Under the Curve (AUC) values of 0.776, 0.779, and 0.822 (3-year, 5-year, and 8-year OS, primary cohort) and 0.658, 0.724, and 0.792 (validation cohort), indicating that the nomogram possessed strong discriminative ability. Calibration and DCA curves further confirmed the nomogram’s predictive accuracy and clinical utility. The nomogram can effectively stratify patients into low-, intermediate-, and high-risk groups, particularly identifying high-risk patients.

**Conclusions:**

The established nomogram in our study can provide precise prognostic information for HCC patients following ablation treatment and enable physicians to accurately identify high-risk individuals and facilitate timely intervention.

## Introduction

Hepatocellular carcinoma (HCC) is revealed to be one of the most prevalent forms of malignancy, resulting in a significant annual mortality rate, claiming the lives of numerous patients each year. The burden of primary liver cancer is particularly severe in China. In China, HCC ranks as the fourth most common type of malignant tumor, with approximately 410,000 new cases and over 390,000 deaths each year (1, 2). The selection of therapeutic strategies for HCC entails a comprehensive evaluation of multiple pivotal factors, encompassing tumor staging, liver function status, and the patient’s overall health (3, 4). For early-stage HCC patients, surgical resection is typically the preferred treatment method, but its actual implementation is often constrained by various factors (5). Causes such as fibrosis, cirrhosis, liver dysfunction, and portal hypertension significantly affect the feasibility and risks of surgical resection. Under these circumstances, local ablation therapy has emerged as a high-profile alternative radical therapy in the treatment of early-stage HCC (6). Nevertheless, for HCC patients who undergo ablation therapy, the lingering predicaments of heightened susceptibility to relapse and an unfavorable long-term prognosis persist, rendering the overall survival of these patients still unsatisfactory (7–9). Therefore, it is crucial to pay attention to the long-term survival of HCC patients after undergoing ablation treatment.

At present, there remains a relative paucity of research regarding the long-term therapeutic efficacy in HCC patients following ablation treatments. Although there have been several pertinent studies, they often come with certain limitations, such as a relatively small sample size or a short follow-up duration (10–12). These limitations result in an insufficient understanding of long-term survival and recurrence risk in patients after treatment. Thus, it is imperative to conduct further large-scale, long-term research, involving a broader patient cohort and more comprehensive data, to achieve a more thorough understanding of the long-term therapeutic outcomes for HCC patients who undergo ablation treatment, ultimately providing improved decision support for doctors. In addition, it is essential to actively embrace cutting-edge technologies, such as machine learning, to bolster research into the long-term treatment outcomes for HCC patients, as they can assist physicians in more effectively analyzing and interpreting clinical data.

This study, incorporating data from nearly 900 patients with a follow-up period exceeding 9 years, combined both traditional statistical methods and machine learning techniques to identify factors that impact the overall survival (OS) of early-stage HCC patients after undergoing ablation therapy. These factors were further visualized in a nomogram for improved assessment of the survival prospects of early-stage HCC patients, aiming to provide clinicians with more precise guidance to improve patients’ treatment outcomes and overall quality of life.

## Materials and methods

This study has received explicit ethical approval from the ethics committee of Beijing You’an Hospital, affiliated with Capital Medical University, which ensured that ethical standards and legal regulations within the research would be strictly adhered to. In light of the study’s retrospective nature, the necessity for obtaining informed consent from patients was exempted.

### Study population

In this study, we collected data from 1342 early-stage HCC patients who underwent ablation therapy and achieved complete response at Beijing You’an Hospital, affiliated with Capital Medical University from January 2014 to December 2022. We then excluded 152 cases of non-primary HCC, 110 cases where ablation therapy was not the initial treatment, 87 cases lacking clinical or follow-up data, 44 cases with distant metastasis, and 68 cases with other infectious or hematologic diseases. Ultimately, 881 cases were included based on the inclusion criteria. Complete ablation response is characterized as the absence of any regions of augmentation either inside the ablated zone or at its periphery one month following the procedure (13). The inclusion criteria were as follows (1): patients were confirmed to have primary HCC through pathological diagnosis and were evaluated at the BCLC 0/A stage; (2) received ablation therapy as primary treatment and obtained complete response; (3) availability of comprehensive clinical and follow-up data. The exclusion criteria were as follows: (1) non-primary tumors. This study was designed to specifically investigate the prognosis of primary HCC tumors after ablation therapy. Non-primary tumors would introduce heterogeneity into the sample, making it difficult to draw firm conclusions about the primary HCC prognosis. (2) patients who have undergone radiotherapy, chemotherapy, or surgical resection before ablation treatment. By excluding these patients, it was ensured that the treatment effect was primarily attributed to the ablation itself, without potential confounding influences from prior therapies. (3) lack of clinical or follow-up data. Excluding patients with incomplete data ensured the reliability and integrity of the study and prevented potential bias caused by missing or incomplete information. (4) patients with observed distant metastasis. The presence of distant metastasis indicated that HCC was in a more advanced stage, but this study focused on early-stage HCC patients who were more suitable for ablation treatment. (5) patients with other infectious or hematological diseases. Coexisting infectious or hematological diseases may impact the immune system and overall health, potentially influencing the prognosis of HCC and response to ablation treatment. Excluding such patients helped in isolating the impact of ablation on HCC without being influenced by additional effects from other medical conditions.

Patients who met the above-qualified criteria were randomly allocated to either the primary or validation cohort through the computer-based randomized number system, maintaining a ratio of 7:3. Ultimately, the primary cohort included data from 609 patients, and the validation cohort included data from 272 patients. The flowchart of the patient’s enrollment and study design is shown in **Supplementary Figure 1**.

### Data collection

In this study, we gathered patients’ baseline characteristic data before ablation treatment and the data encompassed the following categories: (1) personal information: age and gender; (2) medical history: hypertension, diabetes, smoking, and drinking; (3) imaging and pathological features: cirrhosis, Child-Pugh class, Barcelona Clinic Liver Cancer (BCLC) stage, tumor number and tumor size; (4) blood tests: white blood cell (WBC), neutrophil (Neu), lymphocyte (Lym), monocyte (Mon), red blood cell (RBC), hemoglobin (Hb), platelet (PLT), alanine aminotransferase (ALT), aspartate aminotransferase (AST), total bilirubin (TBIL), direct bilirubin (DBIL), albumin (Alb), globulin (Glob), gamma glutamyl transpeptidase (GGT), alkaline phosphatase (ALP), prealbumin (Palb), international normalized ratio (INR), activated partial thromboplastin time (APTT), activated partial thromboplastin time ratio (APTTR), fibrinogen (Fib), thrombin time (TT) and alpha-fetoprotein (AFP).

### Ablation procedure

The radiofrequency ablation (RFA) procedures were performed under the guidance of computed tomography (CT) by physicians with at least 5 years of experience. The following were the steps for RFA: (1) Preoperative positioning. Preoperative fasting for 8 hours, use CT to determine the location, size, and adjacent relationship of the tumor, and develop an appropriate needle insertion path, ablation frequency, and ablation time. (2) Anaesthesia. Routine disinfection and drape in the surgical area, and local anesthesia at the puncture point (intravenous anesthesia can be used for those with poor pain tolerance). (3) Start puncture and ablation. During CT guidance, the radiofrequency electrode needle reached the tumor tissue through the puncture point. The needle insertion was performed in a stepwise manner, adjusting the puncture angle and depth based on the ablation target. After confirming the active end of the radiofrequency electrode needle reached the ablation target through scanning, the needle was fixed in place. The angles and depths of the radiofrequency electrode needle were recorded to prevent displacement during the procedure. During ablation, treatment parameters were set according to the type of radiofrequency ablation device, the model of the electrode needle, the size of the tumor, and its relationship with the surrounding tissue structure. To ensure the effectiveness of tumor ablation therapy, the ablation range should encompass the tumor and 0.5-1.0 cm of surrounding liver tissue. (4) Withdrawing of the needle. Before withdrawing the needle, the needle tract needed to be ablated to prevent bleeding and needle tract seeding. Long-term tracking and monitoring after ablation were crucial links, as patients need to undergo regular follow-up and imaging examinations to continuously monitor tumor recurrence and assess overall survival.

### Follow-up

Overall survival (OS) is a critical endpoint in medical research, which measures the length of time from a specific point, such as the date of ablation treatment in this study, until the date of the patient’s death or last follow-up. After completing the HCC ablation therapy at our hospital, all patients are subsequently followed up one month later, and then at three-month intervals within the first year, followed by semi-annual follow-ups until death or last follow-up. During each follow-up, it typically encompasses radiological examinations such as CT scans or MRIs are conducted to assess the tumor size, location, and any newly discovered anomalies. Furthermore, blood tests are employed to measure liver function and tumor markers, in order to scrutinize any aberrations in biochemical indicators. The last follow-up date for this study was June 30, 2023 and the median follow-up period was 46.5 months.

### Statistical analysis

All statistical analyses in this study were carried out via the R software version 4.2.1. To ascertain the model’s universality, patients were randomly allocated into two groups: a primary group and a validation group, with a ratio of 7 to 3. The primary group served for model development, while the validation group was employed for model verification. Categorical variables are depicted in terms of frequency (percentage) and are subjected to comparison through the χ^2^-test (or Fisher’s exact test, if necessary). Continuous variables are represented as mean ± standard deviation and are subjected to comparison through the *t-*test (or Mann‐Whitney *U* test, if necessary). Least absolute shrinkage and selection operator (Lasso) and multivariate Cox regression techniques were applied to discern independent prognostic variables. Based on the multivariate Cox regression analysis, factors demonstrating p-values less than 0.05 were utilized in the development of the 3-year, 5-year, and 8-year OS nomogram. The performance of the nomogram was evaluated through the concordance index (C-index), receiver operating characteristic (ROC), calibration plot and decision curve analysis (DCA). C-index and ROC curve were utilized to assess the discriminatory power and predictive accuracy of the established nomogram, with values spanning from 0 to 1.0. A value of 0.5 signifies random chance, while a value of 1.0 indicates ideal accuracy in forecasting events. The calibration plot provided a visual representation of the alignment between predicted survival and observed survival by employing a bootstrap method with 1,000 resampling iterations, helping us gauge how well a model’s predictions match reality. DCA curve played a pivotal role in evaluating the clinical utility of the nomogram by quantifying its net benefit under different thresholds. Additionally, in accordance with the total score computed from the nomogram in the primary cohort, the patients were stratified into three categories, specifically, low-risk, intermediate-risk and high-risk groups. Kaplan-Meier survival curves and the log-rank test were then adopted for assessing and comparing the OS of patients among the three groups. The significance tests employed in this study were two-tailed, and statistical significance was defined as a p-value less than 0.05. Furthermore, individuals were stratified into three risk groups—low, intermediate, and high—according to the scores derived from the nomogram. Subsequently, Kaplan-Meier curves were employed to predict the survival rate for each group.

## Results

### Patients’ demographic and clinical characteristics

In this study, we enrolled a grand total of 881 qualified early-stage HCC patients treated by ablation, randomly divided into two cohorts: the primary cohort, which consisted of 609 patients, and the validation cohort, which encompassed 272 patients. **Table 1** displays the patient baseline characteristics within the two cohorts, and it can be seen that there is no statistical significance in all terms of the variables (p > 0.05), which indicates similarity between the cohorts. Among these patients, their mean age was over 55 years old and 710 (80.6%) were male. 237 individuals (26.9%) were diagnosed with hypertension, while 195 individuals (22.1%) were found to have diabetes. 762 (86.5%) cases of patients suffered from cirrhosis, accounting for 86.5% of the total. 722 (82.0%) patients exhibited singular tumor manifestations, while 159 (18.0%) cases were identified with the presence of multiple tumors. 649 (73.7%) cases showed a tumor size<3 cm and 232 (26.3%) presented a tumor size≥3cm. According to the Child-Pugh classification, 669 (75.9%) patients were categorized as class 0 and 212 (24.1%) patients were designated as class A. In the BCLC staging, 330 (37.5%) were categorized as stage 0, while 551 (62.5%) fell into stage A. It should be noted that our analysis mainly revolves around static baseline characteristics. However, variables such as GGT and Palb may change over time. The absence of these dynamic data may limit a comprehensive understanding of patients’ evolving conditions and treatment responses. Future research could prioritize collecting dynamic data to offer a more nuanced exploration of patient’s prognosis.

**Table 1:** Demographic and clinical characteristics of the patients in two cohorts.

Characteristic	Primary cohort(N=609)	Validation cohort(N=272)	P value
Age	56.26 ± 9.13	57.56 ± 9.02	0.151
Gender (male/female)	493(81.0%)/116(19.0%)	217(79.8%)/55(20.2%)	0.684
Hypertension (yes/no)	159(26.1%)/450(73.9%)	78(28.7%)/194(71.3%)	0.427
Diabetes (yes/no)	137(22.5%)/472(77.5%)	58(21.3%)/214(78.7%)	0.699
Antiviral (yes/no)	342(56.2%)/267(43.8%)	162(59.6%)/110(40.4%)	0.346
Smoking (yes/no)	268(44.0%)/341(56.0%)	105(38.6%)/167(61.4%)	0.134
Drinking (yes/no)	201(33.0%)/408(67.0%)	85(31.3%)/187(68.7%)	0.607
Cirrhosis (yes/no)	526(86.4%)/83(13.6%)	236(86.8%)/36(13.2%)	0.875
Tumor number (Single/multiple)	502(82.4%)/107(17.6%)	220(80.9%)/52(19.1%)	0.581
Tumor size (<3cm/≥3cm)	445(73.1%)/164(26.9%)	204(75.0%)/68(25.0%)	0.548
Child-Pugh class(A/B)	468(76.8%)/141(23.2%)	201(73.9%)/71(26.1%)	0.344
BCLC stage (0/A)	225(36.9%)/384(63.1%)	105(38.6%)/167(61.4%)	0.639
WBC	5.11 ± 2.14	4.86 ± 2.01	0.109
Neu	3.23 ± 1.82	3.09 ± 1.67	0.263
Lym	1.33 ± 0.86	1.25 ± 0.63	0.157
Mon	0.42 ± 0.23	0.39 ± 0.22	0.113
RBC	4.19 ± 0.62	4.15 ± 0.64	0.409
Hb	131.50 ± 19.38	130.51 ± 19.77	0.486
PLT	120.91 ± 59.53	122.36 ± 61.71	0.741
ALT	31.31 ± 20.01	31.59 ± 19.63	0.85
AST	31.70 ± 16.19	32.10 ± 13.54	0.722
TBIL	19.35 ± 10.05	19.70 ± 10.15	0.639
DBIL	6.53 ± 4.60	7.02 ± 5.07	0.15
Alb	37.50 ± 4.52	37.35 ± 5.28	0.673
Glob	28.16 ± 5.34	28.14 ± 5.20	0.944
GGT	64.85 ± 53.80	66.38 ± 62.50	0.711
ALP	86.47 ± 34.08	88.54 ± 37.95	0.422
Palb	141.19 ± 57.04	140.56 ± 61.45	0.882
INR	1.11 ± 0.13	1.13 ± 0.14	0.152
APTT	33.66 ± 4.18	33.75 ± 4.91	0.794
APTTR	1.12 ± 0.15	1.13 ± 0.18	0.742
Fib	2.77 ± 0.92	2.77 ± 0.91	0.947
TT	15.88 ± 2.16	15.85 ± 2.31	0.847
AFP	284.36 ± 1472.15	165.1 ± 534.54	0.079

BCLC, Barcelona Clinic Liver Cancer; WBC, white blood cell; Neu, neutrophil; Lym, lymphocytes; Mon, monocyte; RBC, red blood cell; Hb, hemoglobin; PLT, platelet; ALT, alanine aminotransferase; AST, aspartate aminotransferase; TBIL: total bilirubin; DBIL, direct bilirubin; Alb, albumin; Glob, globulin; GGT, gamma-glutamyl transpeptidase; ALP, alkaline phosphatase; Palb, prealbumin; INR, international normalized ratio; APTT, activated partial thromboplastin time; APTTR, activated partial thromboplastin time ratio; Fib, fibrinogen; TT, thrombin time; AFP, alpha fetoprotein.

### Screened risk factors for overall survival

**Figure 1:**
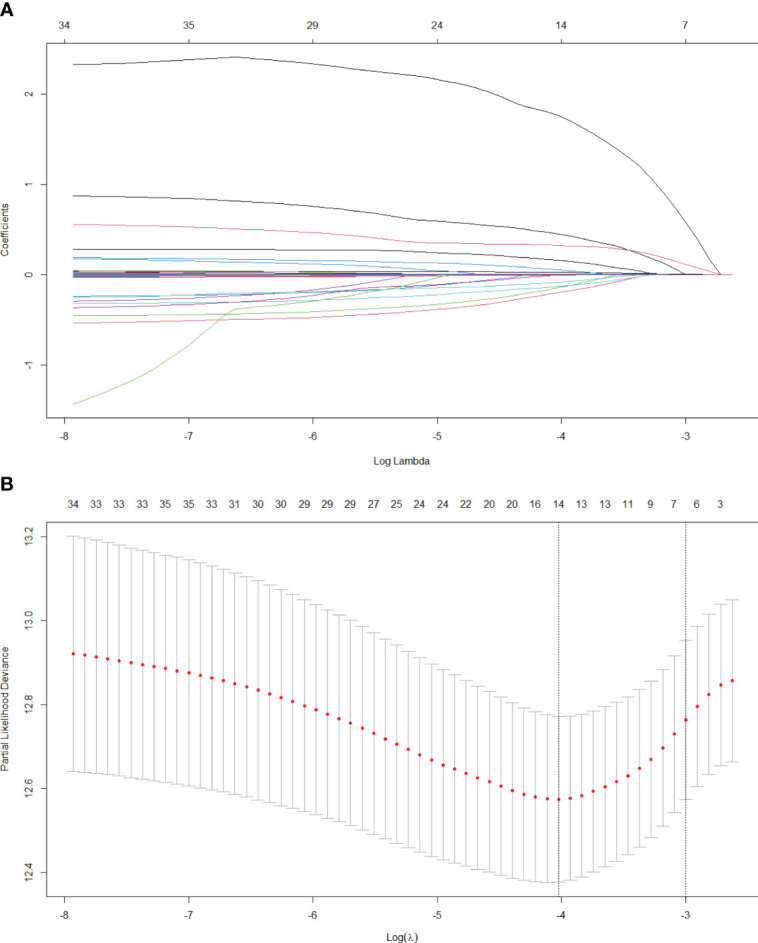
Lasso regression analysis in the primary cohort. **(A)** Variation features of the coefficient of variables; **(B)** Determination of the optimal value of λ through cross-validation method.

**Figure 2:**
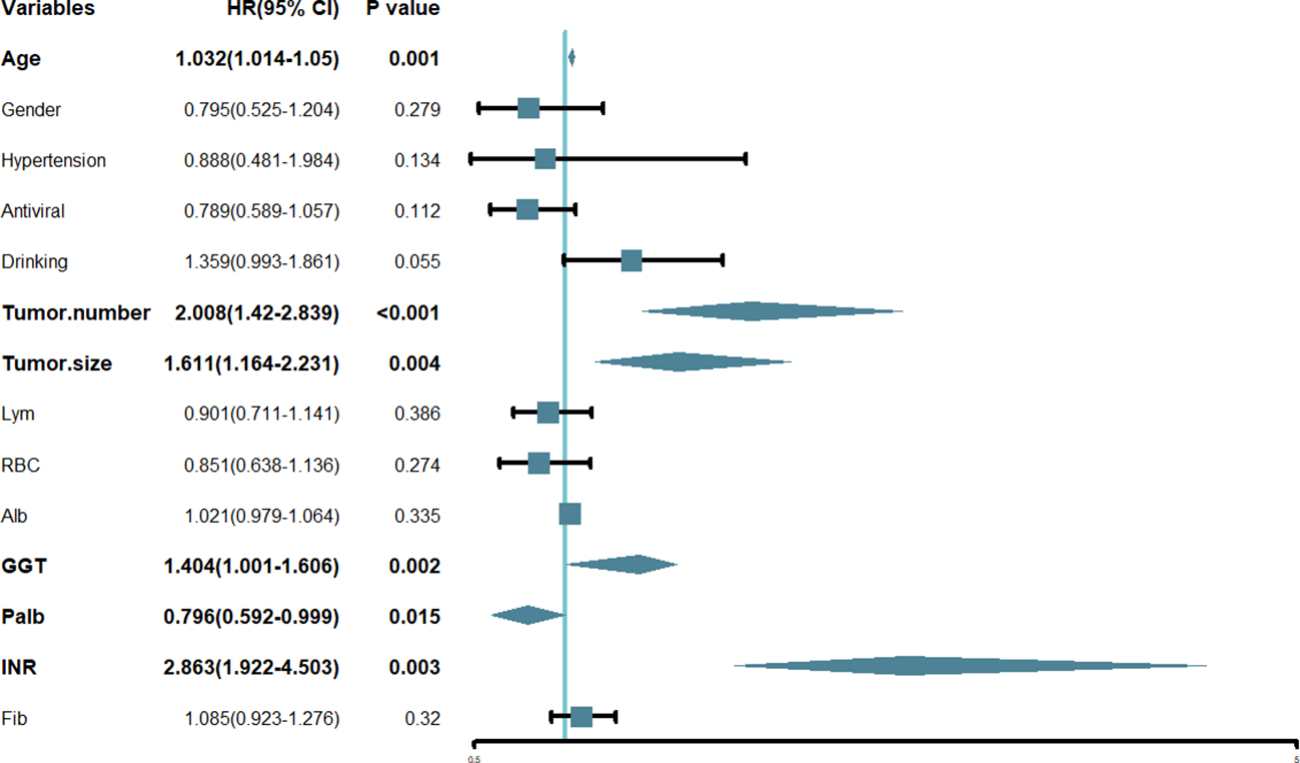
Forest plot of the hazard ratio of the variables based on the multivariate Cox analysis in the primary cohort. HR, hazard ratio; CI, confidence interval.

In this research, we initially employed Lasso regression to select the factors influencing patients’ OS (**Figure 1**). Lasso regression includes a penalty term (i.e. L1 regularization) that enforces shrinkage of some regression coefficients toward zero. The strength of this penalty is controlled by the parameter λ, and its optimal value is commonly confirmed by 10-fold cross-validation. **Figure 1A** is the Lasso regression coefficient path diagram. This study includes 34 variables, so there are 34 lines of different colors. That is, each curve represents the change trajectory of each variable coefficient. The ordinate is the value of the coefficient, the lower abscissa is log(λ), and the upper abscissa is the number of non-zero coefficients in the model at this time. It can be seen that as the log(λ) increases, the regression coefficient (i.e. the ordinate value) continuously converges and eventually converges to 0. **Figure 1B** shows the cross-validation curve of LASSO regression. The lower abscissa is log(λ), and the ordinate is the likelihood deviance. The smaller the ordinate is, the better the fitting effect of the equation is. The upper abscissa is the number of remaining variables in the equation for different λ. In our study, upon reaching a minimum λ value of 0.018, 14 potential predictors linked to OS, with non-zero coefficients, were identified within the primary cohort, which included age, gender, hypertension, antiviral, drinking, tumor number, tumor size, Lym, RBC, Alb, GGT, Palb, INR and Fib. Then, we used the multivariate Cox regression to further identify the most crucial variables essential for OS prediction, and the results were displayed through a forest plot (**Figure 2**). Cox regression is based on a semiparametric model, which assumes that the effect of predictor variables on time of event (death) is described by a hazard proportional function. Therefore, the results of Cox regression are usually presented in the form of hazard ratio (HR). Generally, if the P value is less than 0.05, we consider the result to be significant that there is an association between the predictor variable and the time of event. When the HR value is greater than 1, it indicates that the factor is a promoting factor for the occurrence of the death. If the HR value is less than 1, it indicates that the factor is a hindering factor for the occurrence of the death. If the HR value is equal to 1, it indicates that the factor has no effect on the occurrence of the death. In our analysis, it was revealed that age (HR: 1.032; 95% CI: 1.014 - 1.05; P=0.001), tumor number (HR: 2.008; 95% CI: 1.42 - 2.839; P<0.001), tumor size (HR: 1.611; 95% CI: 1.164 - 2.231; P=0.004), GGT (HR: 1.404; 95% CI: 1.001 - 1.606; P=0.002), Palb (HR: 0.796; 95% CI: 0.592 - 0.999; P=0.015), INR (HR: 2.863; 95% CI: 1.922 - 4.503; P=0.003) were the most significant variables for OS prediction. Of the six variables, age, tumor number, tumor size, GGT, and INR were identified as having a hazardous effect, whereas Palb was considered to have a protective effect.

### Development of the nomogram for overall survival

**Figure 3:**
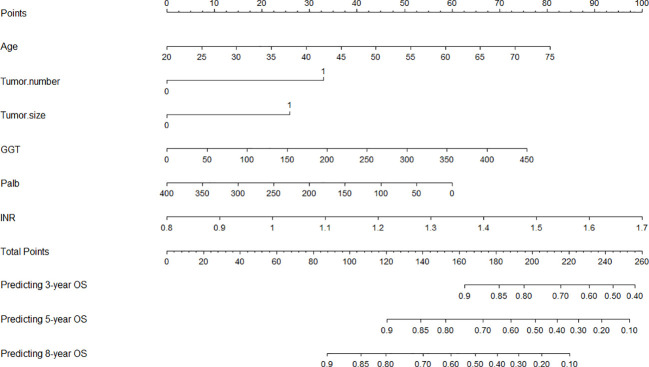
Nomogram for predicting the 3-, 5-, and 8-year overall survival (OS). GGT, gamma-glutamyl transpeptidase; Palb, prealbumin; INR, international normalized ratio.

A nomogram was developed using the six notable prognostic factors identified above, facilitating a visual presentation for predicting 3-year, 5-year, and 8-year OS of early-stage HCC patients who received ablation therapy (**Figure 3**). Each variable was assigned a predictive score, and the cumulative score of the six variables was plotted along the axis for total points, indicating the prognosis for 3-year, 5-year, and 8-year OS probabilities.

### Performance of the established nomogram

**Figure 4:**
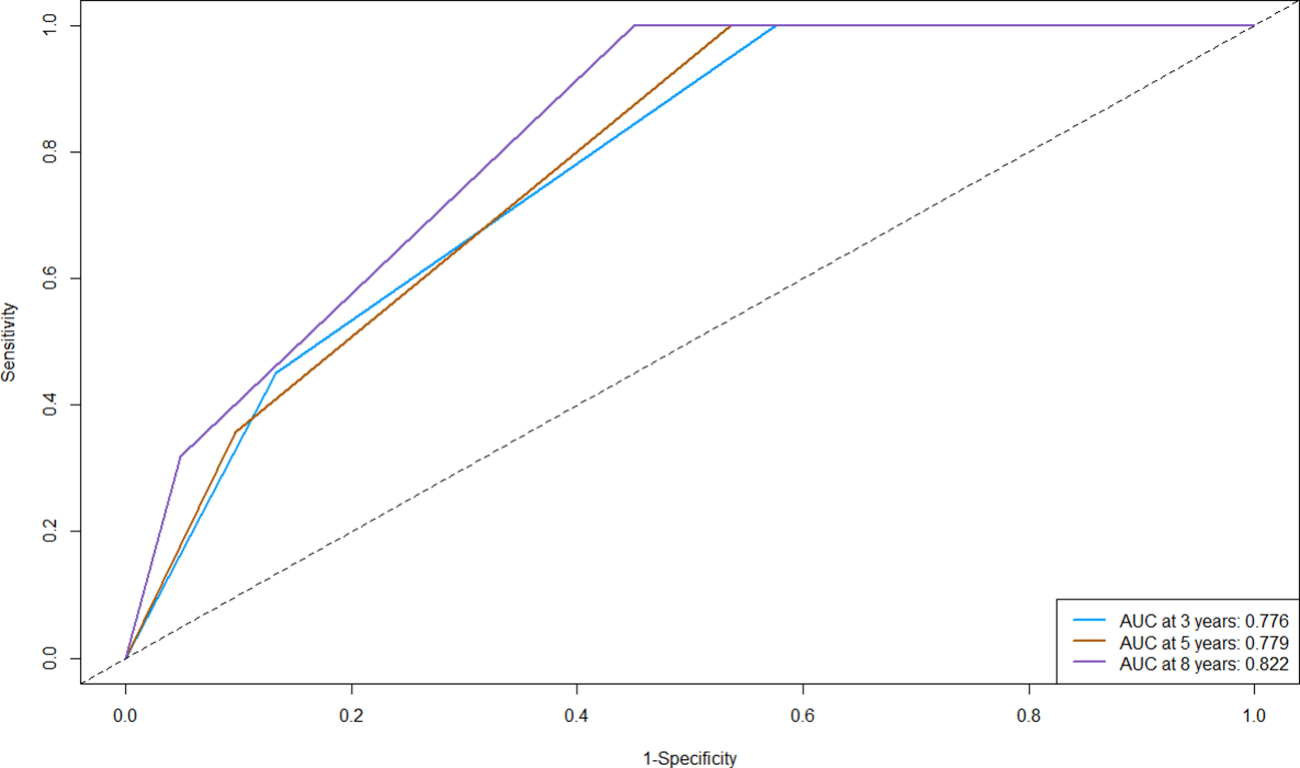
Receiver operating characteristic curve (ROC) of the nomogram for predicting the 3-, 5-, and 8-year overall survival (OS) in the primary cohort. AUC, Area Under the Curve.

C-index is a metric used to quantify the discriminative ability of survival analysis models. The range of the C-index typically falls between 0.5 and 1, where 0.5 indicates that the model’s predictions are no better than random guessing, and 1 signifies perfect prediction. The C-index of the nomogram in the primary cohort is 0.683 (95% CI: 0.636-0.730), indicating that our model was able to well discriminate individuals with different survival times. We proceeded to generate ROC curves for the 3-year, 5-year, and 8-year OS in our primary cohort (**Figure 4**), which was a fundamental tool in machine learning used to evaluate the trade-off between a model’s true positive rate (sensitivity, y-axis) and its false positive rate (1-specificity, x-axis) at various threshold values. The outcomes of our study revealed that the Area Under the Curve (AUC) values corresponding to the 3-year, 5-year, and 8-year OS were 0.776, 0.779, and 0.822, respectively. More specifically, our model exhibited a moderate ability to discriminate between survival and non-survival at the 3-year mark, and its performance improved at the 5-year mark, and reached its highest discriminative potential at the 8-year time point, with an AUC of 0.822. These findings highlight the model’s increasing accuracy in forecasting patient survival as the prediction horizon extends, with the 8-year OS showing the most promising predictive capacity.

**Figure 5:**
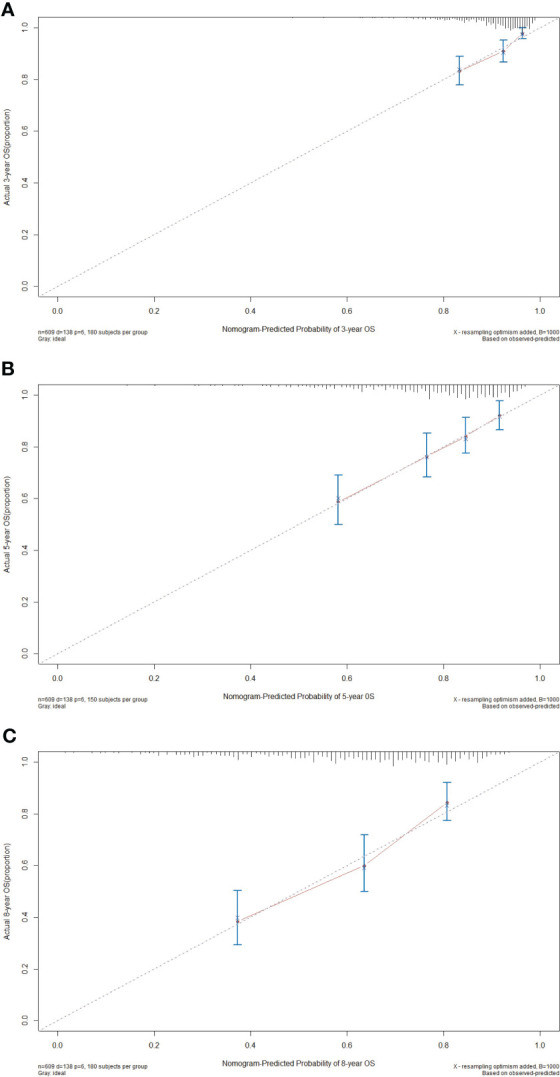
Calibration curves of the nomogram for predicting the 3- **(A)**, 5- **(B)**, and 8-year **(C)** overall survival (OS) in the primary cohort.

Calibration curve serves as a potent instrument for visually contrasting the model’s predictions with real observed data to assess the degree of consistency between them. In the calibration plots, the x-axis typically represents the predicted or measured values, while the y-axis represents the actual or true values. A perfect calibration line follows a 45-degree diagonal from the bottom-left corner to the top-right corner, indicating a perfect match between predicted and actual values. In our study, for the 3-year, 5-year, and 8-year OS probabilities, the calibration curves elucidated an exceptional level of harmony and conformity between the observed clinical outcomes and nomogram-derived survival probabilities (**Figure 5**). The nomogram’s ability to consistently generate dependable survival forecasts at different time points bolsters its position as an invaluable asset in the realm of personalized patient care.

**Figure 6:**
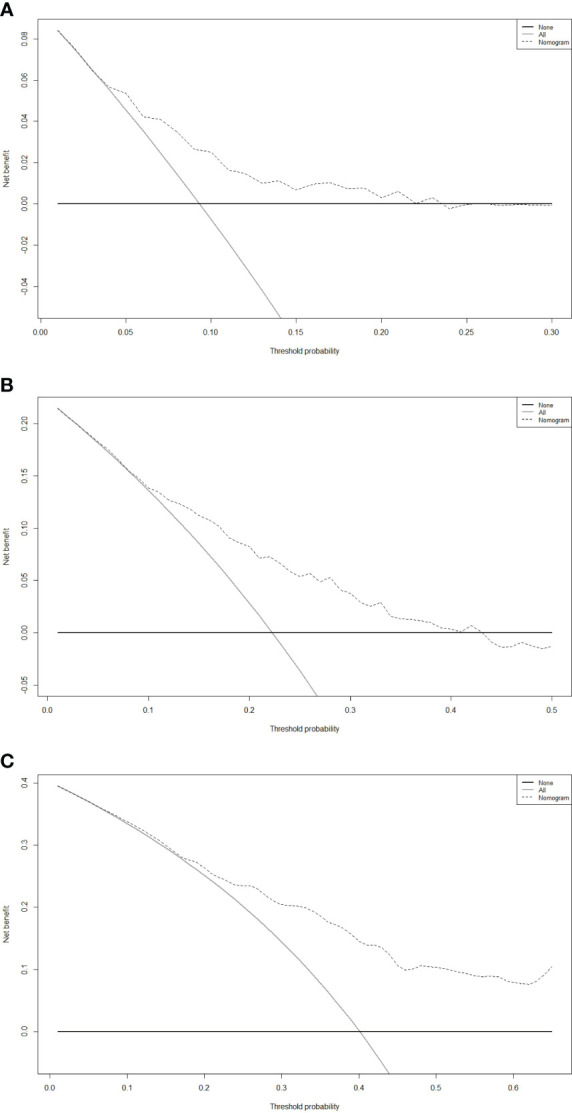
Decision curve analysis (DCA) of the nomogram for predicting the 3- **(A)**, 5- **(B)**, and 8-year **(C)** overall survival (OS) in the primary cohort.

DCA curve plays a pivotal role in clinical practice by quantifying the clinical utility of predictive models. Its role is to evaluate and compare the net benefit of these models across various threshold probabilities, aiding in the selection of the most effective tools for guiding treatment decisions. On the graph, the X-axis represents the threshold probability, which signifies the likelihood at which a physician or healthcare provider would consider using the nomogram’s predictions to make clinical decisions. Meanwhile, the Y-axis depicts the net benefit experienced by the patients when following the recommendations of the nomogram. In this study, our DCA results revealed that the nomogram exhibited substantial net benefits within an appropriate range of threshold probabilities concerning the 3-, 5- and 8-year OS (**Figure 6**). This ability to provide substantial net benefits further signified that the nomogram could aid in making more informed and, ultimately, more beneficial decisions across a wide range of clinical scenarios.

### Validation of the nomogram

To bolster the nomogram’s robustness, we executed an internal validation procedure within the validation cohort. Through this rigorous process, we scrutinized the nomogram’s performance, validating its ability to reliably provide accurate predictions and stand up to the demands of practical clinical applications. The results of this internal verification were quite promising. This validation cohort yielded a C-index of 0.652, accompanied by a 95% confidence interval ranging from 0.579 to 0.725, indicating a reasonable level of discriminatory accuracy. The AUC values for 3, 5, and 8-year OS were notably robust, with scores of 0.658, 0.724, and 0.792, respectively (**Supplementary Figure 2**), which signified the nomogram’s capability to accurately distinguish between individuals who will survive for a specified time-frames and those who will not. Such results were pivotal in affirming the nomogram’s predictive accuracy. The calibration curves for 3-, 5- and 8-year OS demonstrated that the nomogram’s predictions were in close agreement with the actual outcomes (**Supplementary Figure 3**), reaffirming its high degree of consistency, while the 3-, 5- and 8-year DCA curves further underscored its exceptional clinical utility (**Supplementary Figure 4**). It is worth noting that the study’s dependence on data from a single center in China highlights constraints in its generalizability, and it is important to conduct external validation in the future. Furthermore, it is essential to undertake prospective randomized controlled trials in the future to affirm and bolster these findings.

### Prognostic stratification of the patients based on the nomogram scores

**Figure 7:**
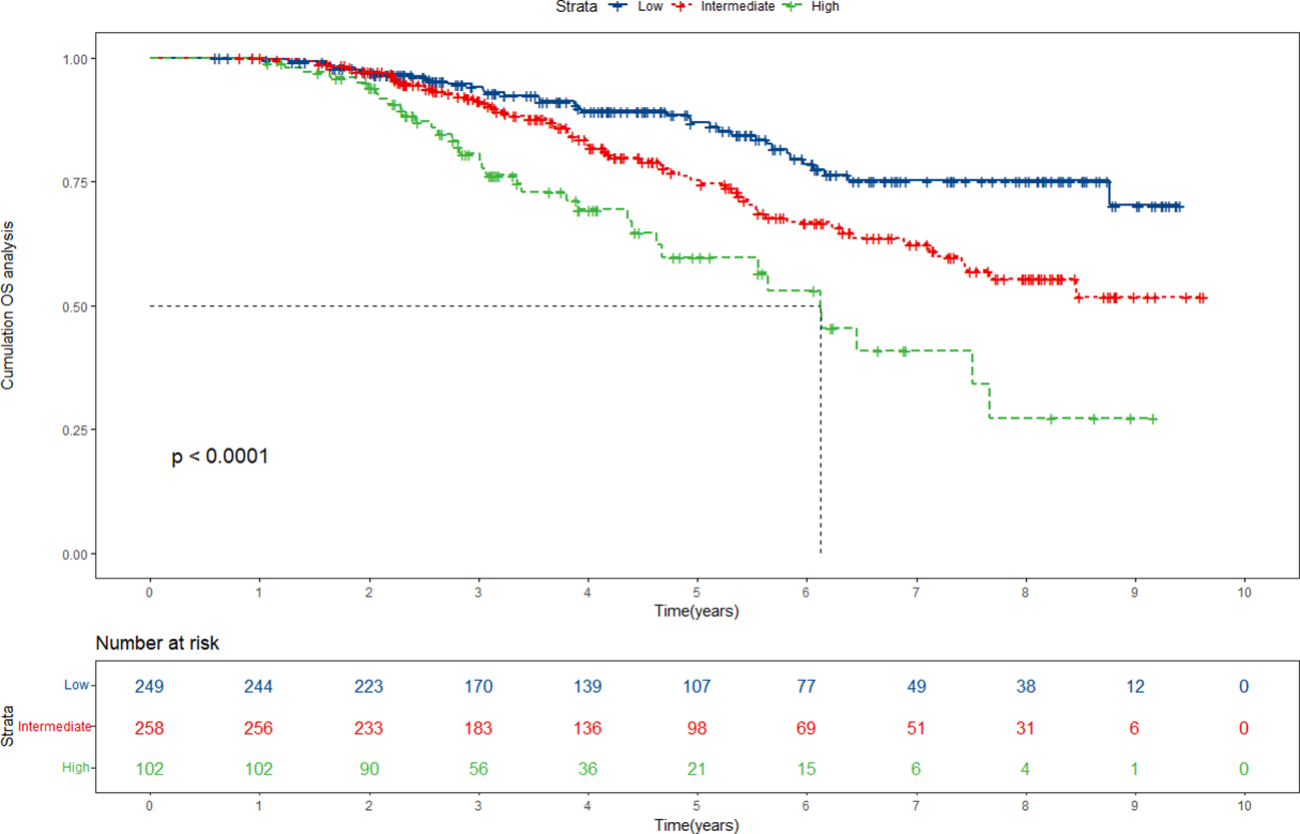
Risk stratification for overall survival (OS) based on the nomogram risk scores in the primary cohort.

Drawing upon the cumulative scores derived from the nomogram, we have established a comprehensive risk stratification system, classifying patients into three distinct risk categories: low-risk, intermediate-risk, and high-risk. Kaplan-Meier analysis unfurled the profound implications of our stratification system. The results revealed a significant divergence in survival probabilities among the three risk groups. Within the primary cohort, patients positioned within the low-risk group experience significantly higher survival rates, while those situated within the high-risk group confront a graver prognosis (**Figure 7**). This divergence was indicative of the nomogram’s effectiveness in accurately identifying patients at higher risk of adverse outcomes. It allowed for the tailoring of interventions to suit the individualized needs of each patient, providing the opportunity for improved outcomes for those facing a high-risk scenario. Significantly, our validation cohort analysis corroborated and mirrored the findings observed in the primary cohort (**Supplementary Figure 5**). The replication of consistent outcomes in the validation cohort further strengthened the reliability and robustness of our nomogram model and risk stratification system, highlighting its versatility and applicability across different patient populations. However, what requires attention is that the nomogram is primarily just applicable to early-stage HCC patients undergoing ablation therapy. Its applicability in cases of advanced HCC or in individuals choosing alternative treatments awaits further study.

### Subgroup analysis

In order to explore the role of the nomogram in subgroups, we performed further analysis, mainly including gender and AFP subgroups. The results of our gender subgroup analysis are shown in **Supplementary Figure 6**. In both the primary and validation cohorts, the nomogram can effectively distinguish the survival risk of male patients with statistically significant differences. However, in females, neither group was significantly differentiated, probably due to the sample size. Indeed, this points to an essential applicability of our model in predicting the OS of male patients. Similarly, as presented in **Supplementary Figure 7**, the nomogram can accurately forecast the prognosis of AFP-positive patients, either in the primary cohort or the validation cohort.

## Discussion

In this study, we meticulously evaluated the baseline demographic and clinical characteristics of patients using a combination of Lasso and multivariate Cox regression techniques. Subsequently, we developed and validated a prognostic nomogram tailored to forecast the 3-, 5-, and 8-year OS rates for early-stage HCC patients who underwent ablation therapy. This nomogram exhibited significant potential in facilitating precise prognosis evaluation and improving the efficiency of post-treatment patient care.

Compared to prior studies investigating the prognostic outcomes of early-stage HCC patients following ablation therapy, this study has some notable advantages. The first significant advantage of this research is the scale of the sample cohort. We have collected data from a large cohort of early HCC patients who underwent ablation therapy, including clinical information, pathological features, and survival data. Having such a massive sample cohort enables us to analyze the differences between different patient groups more accurately, thus enhancing the reliability of the model. A large-scale sample cohort also helps reduce the risk of model overfitting, making our predictive results more general and credible. During the data collection process, we took a series of measures to ensure the quality and accuracy of the collected data. We have established a unified data collection standard, including data collection time, data sources, data presentation methods and so on to ensure data accuracy. We then performed data verification, including checking data consistency, outliers, and missing data. Data cleaning was performed when necessary, including identifying and removing abnormal or unreasonable data and filling in missing or erroneous data. We also conduct quality assessment of data through data sampling and random audits to identify potential problems and take timely corrective measures. The second advantage is that our research comes with long-term follow-up data. Understanding the long-term survival of patients post-treatment is crucial for guiding treatment decisions. Our research team has conducted years of follow-up to ensure that we can capture the long-term effectiveness of patients after ablation therapy. The third advantage lies in our integration of machine learning with traditional statistics. Machine learning techniques can effectively handle large-scale data and discover complex associations hidden within the data. We utilized machine learning, specifically Lasso regression, to uncover potential predictive factors and combined it with traditional statistical methods like multivariate Cox regression to establish a more accurate survival model. This integrated approach enables us to better understand the intricate relationships between a patient’s survival and various factors, providing a more solid foundation for personalized treatment.

Various staging systems have been developed by previous researchers to enhance the assessment of survival prognosis in HCC patients, such as China Liver Cancer Staging (CNLC), Chinese University Prognostic Index (CUPI), Cancer of the Liver Italian Program (CLIP) score, BCLC staging system, the eighth edition of American Joint Committee on Cancer (AJCC) staging manual, Okuda staging system, the Japan Integrated Staging score (JIS score) and so on, many of which have been used in related prediction analysis. However, many of these established systems often fall short in addressing the intricacies of specific HCC treatment methods, emphasizing the necessity for a more nuanced evaluation framework. To the best of our knowledge, our nomogram was the first to simultaneously incorporate the following six factors, namely, age, tumor number, tumor size, GGT, Palb and INR, to predict the OS of early-stage HCC patients after receiving ablation therapy. These factors can be easily acquired through routine clinical examinations, reducing additional costs and patient burdens. Our nomogram not only considers traditional factors such as age and tumor characteristics (tumor number and tumor size) but also incorporates biomarkers like GGT and INR (provide a more comprehensive assessment of liver function), as well as Palb (reflects the overall metabolic status of the patient). Thus, our nomogram stands as a pioneering effort to address the limitations of existing staging systems and provide a more comprehensive and personalized tool for predicting the prognosis of HCC patients.

In this study, we explicitly identified age as a critical risk factor for predicting the overall survival of early-stage HCC patients following ablative therapy. With increasing patient age, the overall survival significantly decreases. Although previous studies have indicated that age may have a significant impact on survival prediction in HCC patients (14–17), our study provides deeper insights through a larger patient sample and more detailed analysis, offering more specific data support for medical practice. The tumor number has also been confirmed to worsen the prognosis of HCC patients, consistent with previous research. The increase in the number of tumors is usually accompanied by more liver function impairment, and ablative therapy typically damages a certain amount of normal liver tissue. If a patient has multiple lesions, it may lead to extensive liver damage, potentially resulting in deteriorating liver function and an increased risk of postoperative complications. Moreover, the presence of multiple tumor nodules may indicate genetic heterogeneity in HCC (18–20). Different tumor nodules may have different genetic variations, which can lead to varying responses to treatment. This genetic heterogeneity makes some tumor nodules more tolerant to ablative therapy, thereby reducing the success rate of treatment. Tumor size is another prognostic risk factor for HCC patients. Our research showed that patients with tumors smaller than 3cm had a longer overall survival than those with tumors larger than 3cm. Many scholars have already found that patients with small HCC tumors have a better prognosis (21–23), including those who have undergone treatments such as surgical resection, ablation, TACE, and so on. Larger HCC tumors may be more prone to invade surrounding tissues, making treatment more complex, and the outcomes may not be as favorable as with smaller tumors. In addition, larger tumors may be more susceptible to local recurrence after treatment. Even in situations where complete ablation appears after treatment, small residual tumor cells in large tumors may still exist, and these cells may regrow after a period of time, leading to tumor recurrence.

The relevant indicators of liver function, including GGT and INR, are also included in the nomogram model of this study. GGT is an enzyme located on the cell membrane, primarily involved in glutathione metabolism and amino acid transport (24). GGT may act as an oxidative stress amplifier, and persistent oxidative stress stimulation can lead to the destabilization of gene stability, disrupting the balance between cell proliferation and apoptosis, thereby affecting tumor formation and progression (25, 26). Moreover, the elevation of GGT may indicate increased invasiveness in HCC as it is involved in the survival, proliferation, and migration of tumor cells, thereby promoting tumor spread and growth. Furthermore, researchers have indicated that certain inflammatory cytokines can induce the expression of GGT (27). Therefore, GGT may be closely associated with inflammation responses related to tumors. This suggests that GGT not only plays a role in the growth and spread of tumors but may also be involved in regulating immune and inflammatory responses associated with tumor development. INR is a standardized index used to measure coagulation function. Multiple research studies have consistently revealed a strong correlation between elevated INR values and unfavorable outcomes among patients diagnosed with HCC (28, 29). A high INR reading typically signifies substantial coagulation dysfunction in patients, consequently elevating the risk of bleeding during and after interventions. An elevated INR value can be attributed to impaired liver function in patients, which in turn, reduces their chances of survival. Moreover, it is intricately linked to the nutritional status and overall health of HCC patients, who frequently experience weight loss, anemia, and malnutrition.

Palb served as a protective factor in our study. Low Palb levels may have a negative impact on the overall survival of early-stage HCC patients after receiving ablation therapy. Past research has shown a close association between the decline in prealbumin levels and the development and progression of HCC (30, 31). HCC patients often present with impaired liver function, and Palb serves as a crucial indicator of hepatic functionality. Thus, a reduction in Palb levels may indicate compromised liver function. Furthermore, HCC patients frequently face the risk of malnutrition due to factors such as tumor compression and metabolic disruptions, leading to a decrease in Palb levels. Therefore, low Palb levels may reflect the overall deterioration of the physical condition of HCC patients (32).

The nomogram we established is an intuitive tool that integrates various predictive factors into a visual chart, enabling clinicians to quickly assess a patient’s survival expectations. By identifying patients’ survival risks in advance, clinicians can choose treatment options more specifically. For example, for high-risk patients, they may be more inclined to choose treatments that focus more on therapeutic effects, while for low-risk patients, more emphasis may be placed on reducing treatment-related adverse events. This personalized treatment selection helps maximize the effectiveness of treatment and reduce unnecessary complications for patients. In addition, the use of this nomogram can also provide guidance for clinical follow-up. Based on the survival risk predicted by the nomogram, clinicians can develop a more reasonable follow-up plan and monitor high-risk patients more frequently to detect and timely intervene in potential problems. This not only helps improve patient survival rates but also effectively utilizes medical resources and reduces the burden on the medical system.

It is important to acknowledge that our study exhibits several deficiencies. To begin with, this study was conducted retrospectively and the nomogram’s development and validation relied solely on data from a single center in China, which may limit its persuasiveness to a broader population. Therefore, it is imperative to conduct external validation as well as prospective randomized controlled trials in the future in order to substantiate and confirm our findings. Second, the results of this study are primarily applicable to early-stage HCC patients undergoing ablation therapy, and further research is needed to assess their applicability to other populations or treatments. For example, while our study does not focus on second-line therapies, immunotherapy, or antiangiogenesis, these treatments become crucial in managing HCC patients with recurrence or progression beyond ablation (33). Our nomogram’s predictive power can aid treatment decisions by identifying higher-risk patients and guiding the selection of appropriate therapeutic strategies, informing our consideration of exploring these treatments in future studies. Third, our research did not deeply explore the impact of socioeconomic status and lifestyle factors on HCC prognosis due to the limited availability of pertinent information within the hospital’s medical record system. The integration of such data in future studies could contribute to a more holistic assessment of the factors influencing HCC outcomes, ultimately enhancing the quality and applicability of our findings in clinical practice. Fourth, our research primarily concentrated on demographic and clinical factors. Some researchers have already initiated studies on the impact of molecular biological factors on cancer (34). Therefore, incorporating these relevant factors in the future could enhance the depth of our predictive model. Finally, this study only included the baseline characteristics of patients, while GGT and Palb, these indicators, are subject to dynamic changes. Therefore, in future research, it could be considered to collect dynamic data of the relevant indicators to gain a deeper understanding of the patients’ condition and treatment response.

## Conclusion

The established nomogram in our study can provide precise prognostic information for HCC patients following ablation treatment and enable physicians to accurately identify high-risk individuals and facilitate timely intervention.

## Data availability statement

The original contributions presented in the study are included in the article/**Supplementary Material**. Further inquiries can be directed to the corresponding authors.

## Ethics statement

The studies involving humans were approved by ethics committee of Beijing You’an Hospital, affiliated with Capital Medical University. The studies were conducted in accordance with the local legislation and institutional requirements. The ethics committee/institutional review board waived the requirement of written informed consent for participation from the participants or the participants’ legal guardians/next of kin because In light of the study’s retrospective nature, the necessity for obtaining informed consent from patients was exempted.

## Author contributions

HZ: Conceptualization, Formal Analysis, Methodology, Writing – original draft, Writing – review & editing. SS: Data curation, Formal Analysis, Writing – original draft, Writing – review & editing. WQ: Data curation, Formal Analysis, Writing – original draft, Writing – review & editing. MH: Conceptualization, Methodology, Project administration, Writing – review & editing. RJ: Conceptualization, Funding acquisition, Methodology, Project administration, Writing – review & editing.

## Supplementary material

Supplementary Figure 1 Flowchart of the patients enrollment and study design.

Supplementary Figure 2 Receiver operating characteristic curve (ROC) of the nomogram for predicting the 3-, 5-, and 8-year overall survival (OS) in the validation cohort. AUC, Area Under the Curve.

Supplementary Figure 3 Calibration curves of the nomogram for predicting the 3- **(A)**, 5- **(B)**, and 8-year **(C)** overall survival (OS) in the validation cohort.

Supplementary Figure 4 Decision curve analysis (DCA) of the nomogram for predicting the 3- **(A)**, 5- **(B)**, and 8-year **(C)** overall survival (OS) in the validation cohort.

Supplementary Figure 5 Risk stratification for overall survival (OS) based on the nomogram risk scores in the validation cohort.

Supplementary Figure 6 Gender subgroup analysis in the primary and validation cohorts. **(A)** Kaplan-Meier curves of male in the primary cohort. **(B)** Kaplan-Meier curves of female in the primary cohort. **(C)** Kaplan-Meier curves of male in the validation cohort. **(D)** Kaplan-Meier curves of female in the validation cohort.

Supplementary Figure 7 AFP subgroup analysis in the primary and validation cohorts. **(A)** Kaplan-Meier curves of AFP-positive in the primary cohort. **(B)** Kaplan-Meier curves of AFP-negative in the primary cohort. **(C)** Kaplan-Meier curves of AFP-positive in the validation cohort. **(D)** Kaplan-Meier curves of AFP-negative in the validation cohort.
