# Supplementary figures and images for: A novel nomogram to predict the overall survival of early-stage hepatocellular carcinoma patients following ablation therapy
Source: Front Oncol. 2024 Feb 7;14:1340286. doi: 10.3389/fonc.2024.1340286 (PMC10880021; doi:10.3389/fonc.2024.1340286)

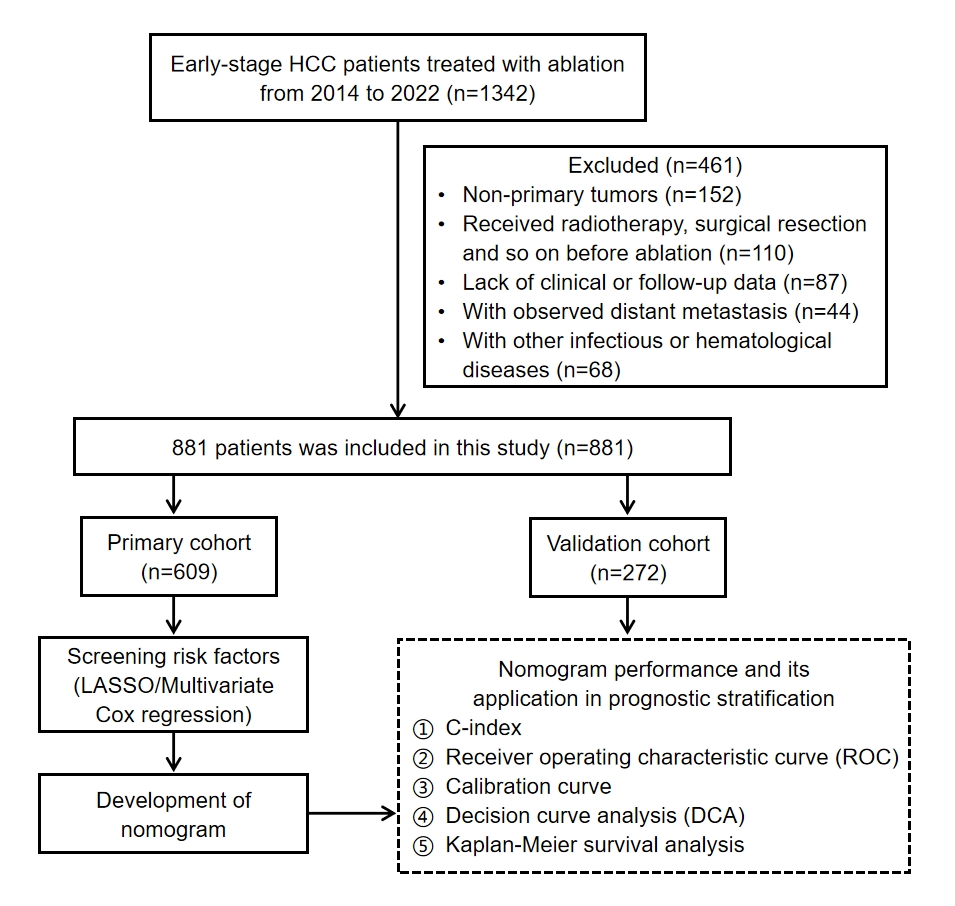

Supplement: Supplementary Figure 1 — Flowchart of the patients enrollment and study design. [file Image_1.jpeg]

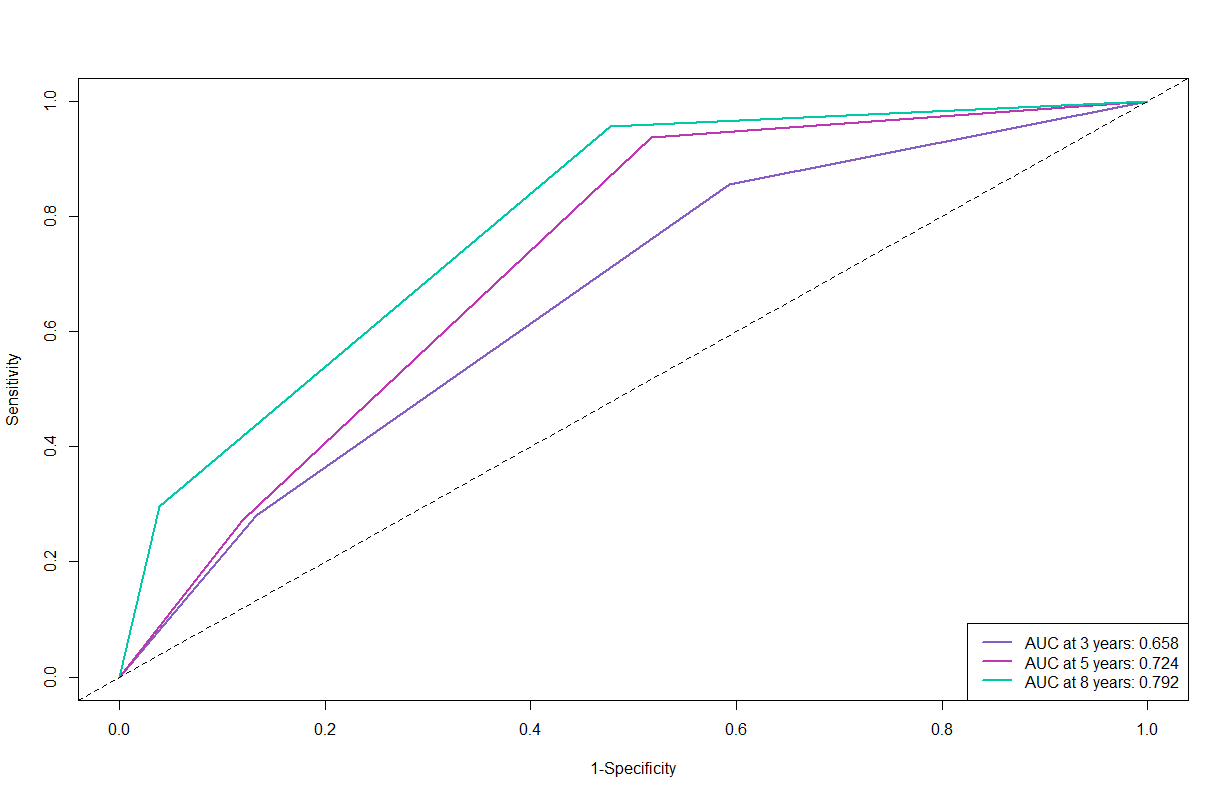

Supplement: Supplementary Figure 2 — Receiver operating characteristic curve (ROC) of the nomogram for predicting the 3-, 5-, and 8-year overall survival (OS) in the validation cohort. AUC, Area Under the Curve. [file Image_2.tiff]

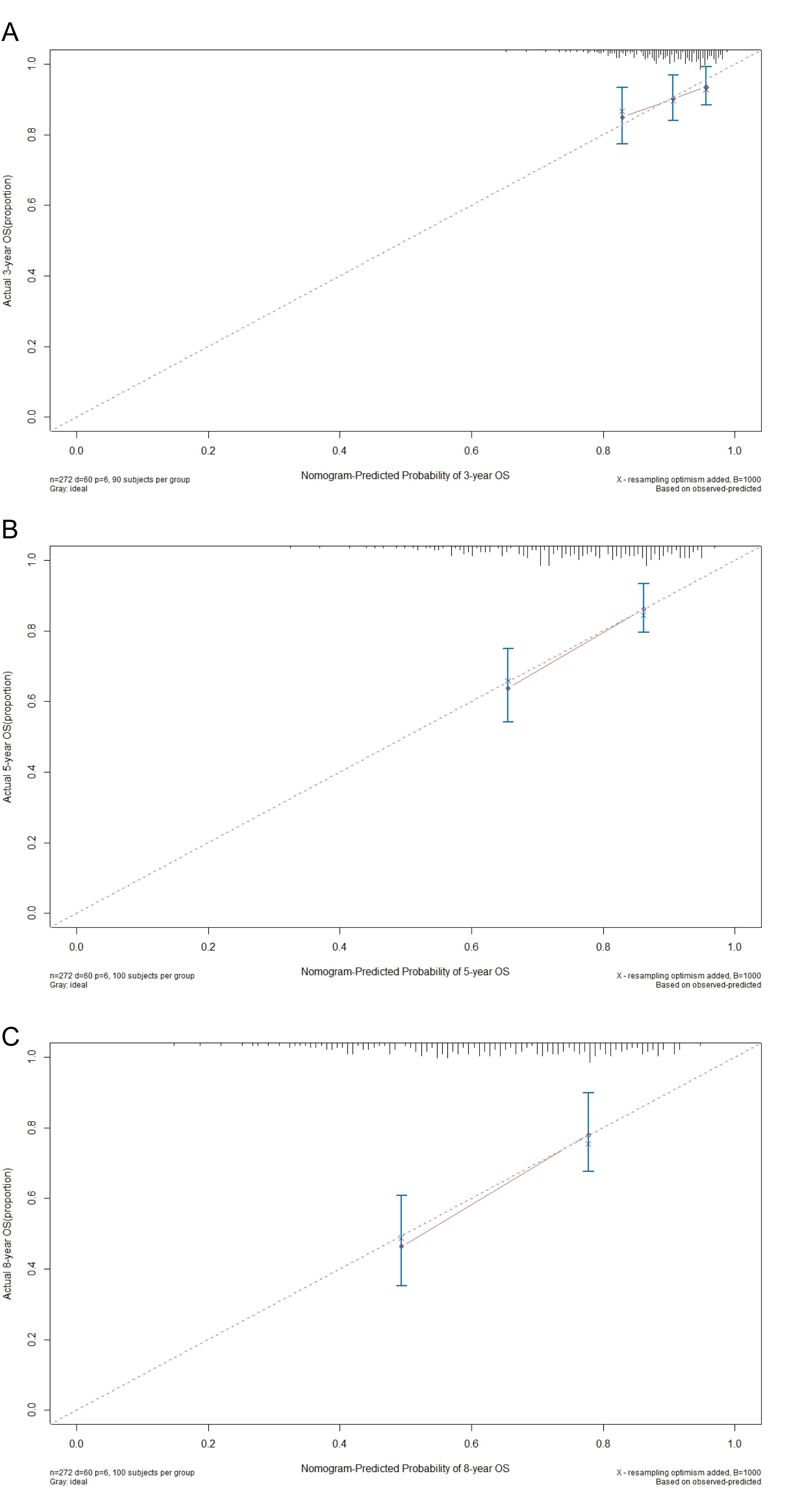

Supplement: Supplementary Figure 3 — Calibration curves of the nomogram for predicting the 3- (A), 5- (B), and 8-year (C) overall survival (OS) in the validation cohort. [file Image_3.jpeg]

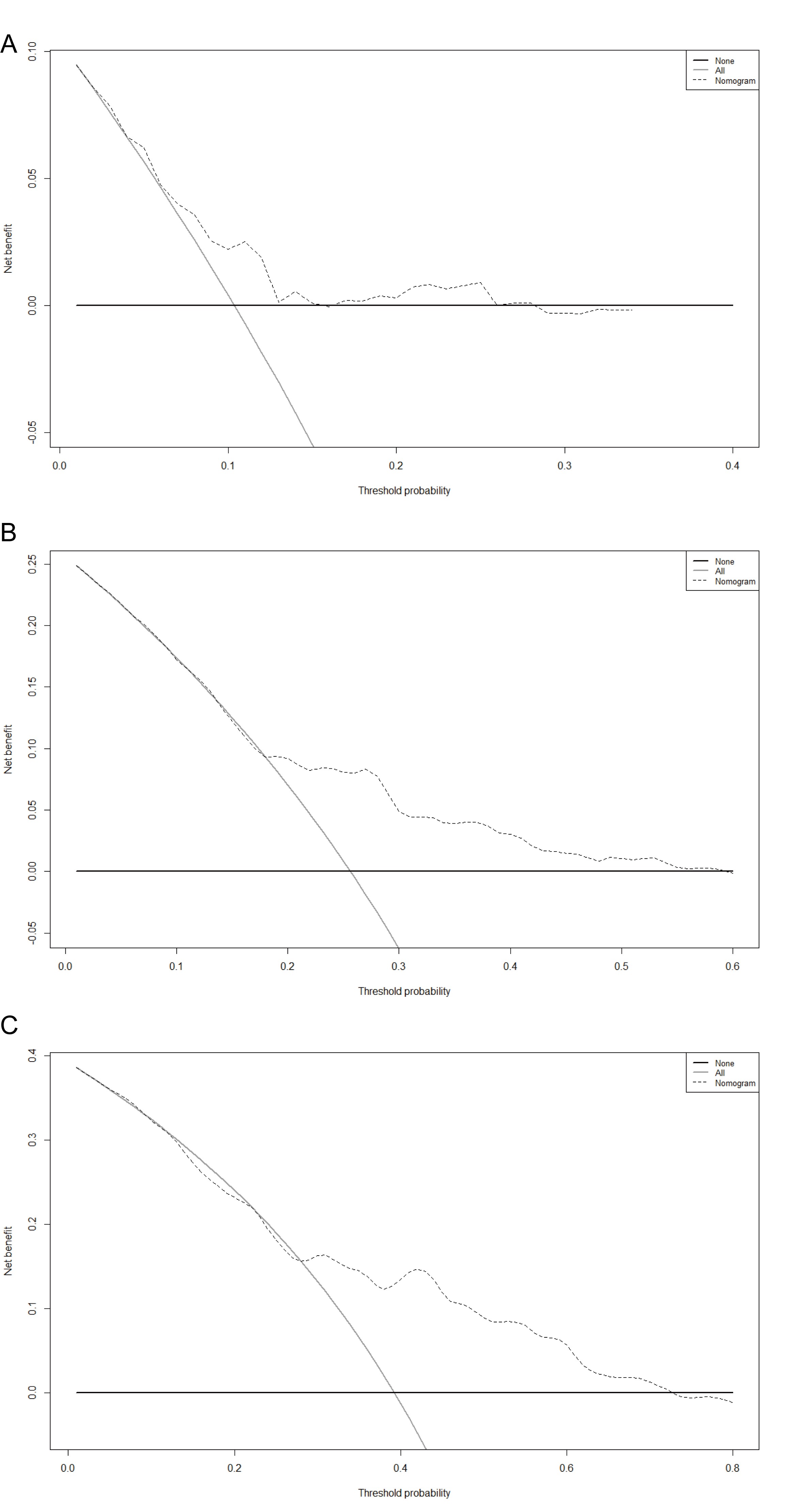

Supplement: Supplementary Figure 4 — Decision curve analysis (DCA) of the nomogram for predicting the 3- (A), 5- (B), and 8-year (C) overall survival (OS) in the validation cohort. [file Image_4.jpeg]

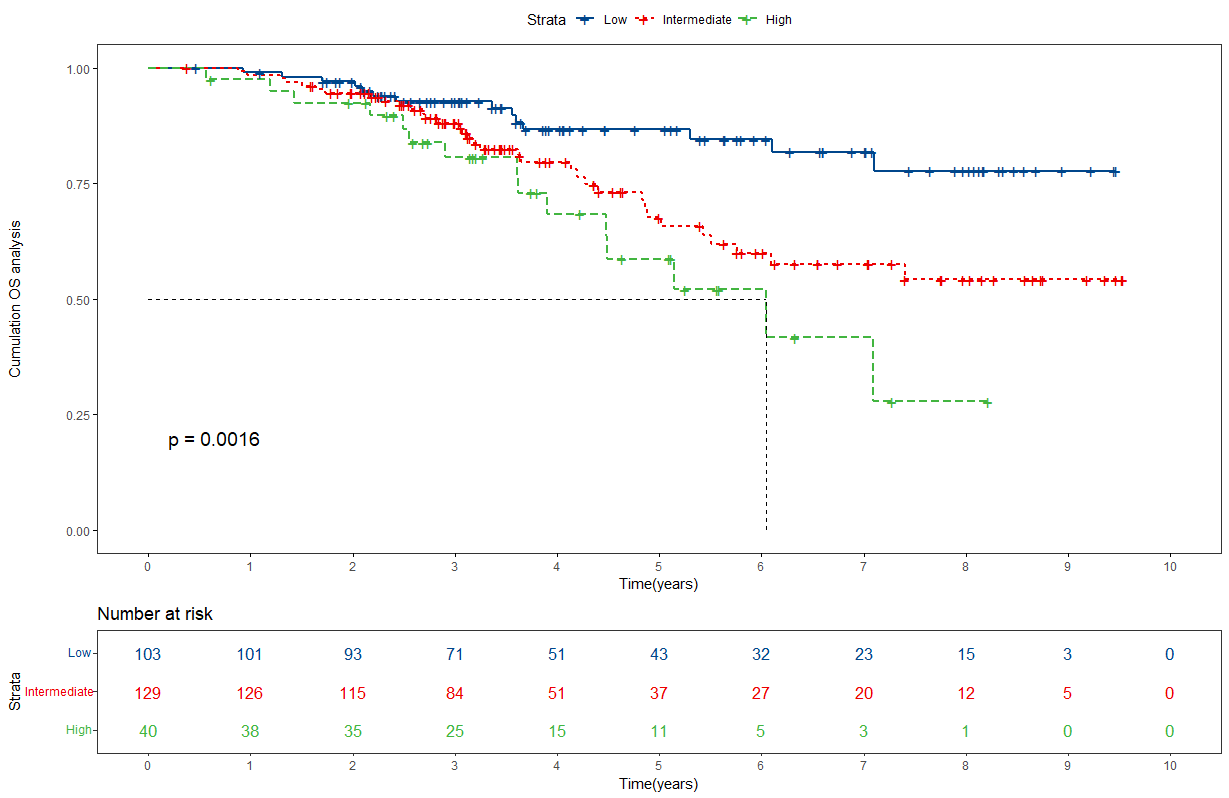

Supplement: Supplementary Figure 5 — Risk stratification for overall survival (OS) based on the nomogram risk scores in the validation cohort. [file Image_5.tiff]

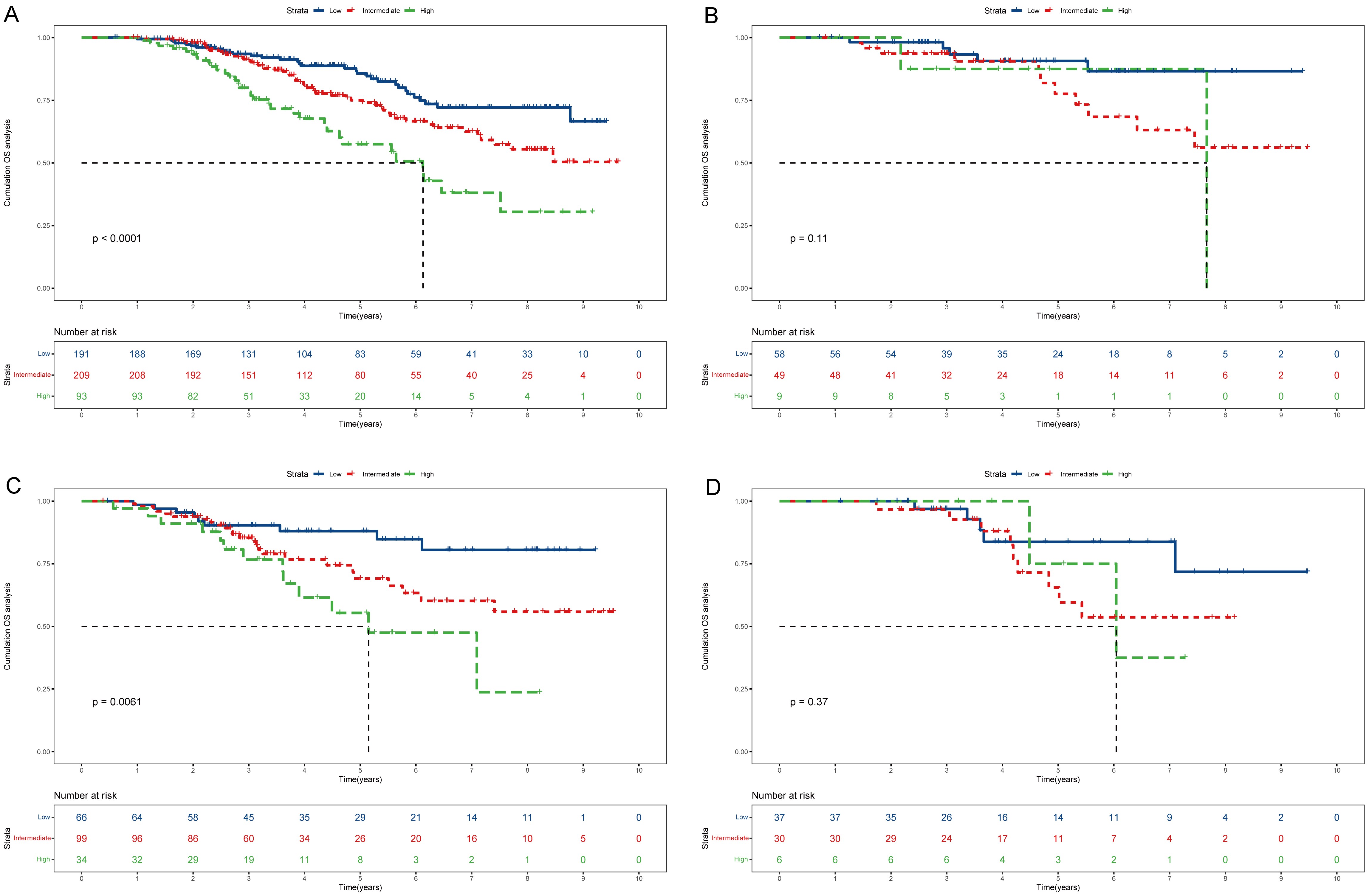

Supplement: Supplementary Figure 6 — Gender subgroup analysis in the primary and validation cohorts. (A) Kaplan-Meier curves of male in the primary cohort. (B) Kaplan-Meier curves of female in the primary cohort. (C) Kaplan-Meier curves of male in the validation cohort. (D) Kaplan-Meier curves of female in the validation cohort. [file Image_6.jpeg]

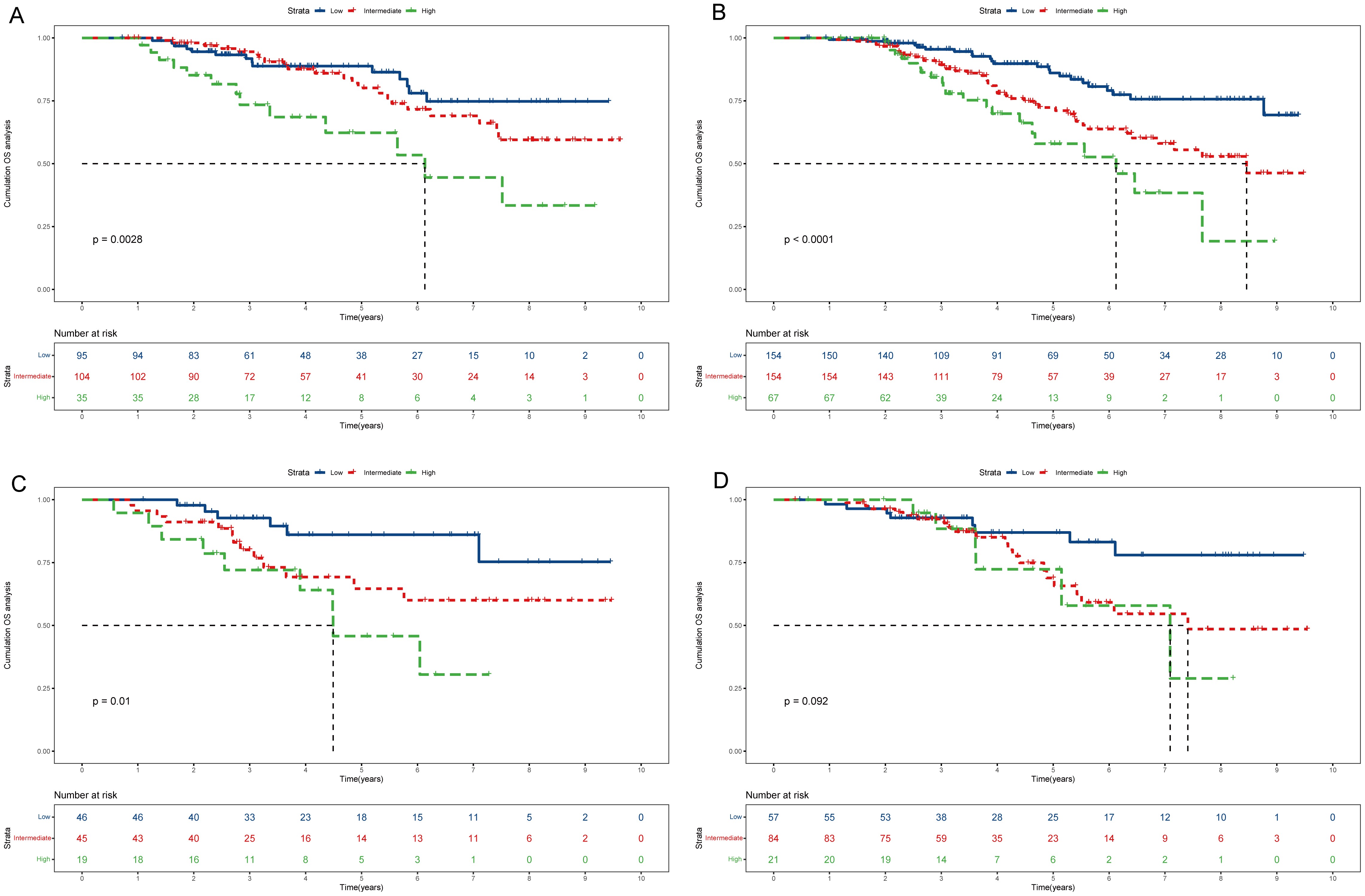

Supplement: Supplementary Figure 7 — AFP subgroup analysis in the primary and validation cohorts. (A) Kaplan-Meier curves of AFP-positive in the primary cohort. (B) Kaplan-Meier curves of AFP-negative in the primary cohort. (C) Kaplan-Meier curves of AFP-positive in the validation cohort. (D) Kaplan-Meier curves of AFP-negative in the validation cohort. [file Image_7.jpeg]
